# Artificial Lighting for Public Institutions

**Published:** 1898-11-26

**Authors:** Carlton Lambert

**Affiliations:** Royal Naval College, Greenwich.


### The Institutional Workshop.

### ARTIFICIAL LIGHTING FOR PUBLIC INSTITUTIONS.

### GAS AND ELECTRIC.

By Professor Carlton Lambert, M.A., Royal Naval College, Greenwich.

The importance of a system of artificial lighting which shall be at once efficient, economical, and healthy, renders a study of the relative advantages of the two illuminants, gas and electricity, in the light of modern developments, of great interest to those who are concerned in the working of our public institutions.

Were it not for the invention of Carl Auer von Welsbach, who, in 1887, taught us how to obtain from a cubic foot of gas eight times as much light as we had been accustomed to from our open burners, gas as an illuminant would have been almost entirely out of the field at the present day.

The Welsbach mantle has effected a great revolution in artificial lighting, and has made gas absolutely unapproachable at present by its competitors as a cheap illuminant. It consists, as is well known, of a delicate network of rare earth, thoria, with a 1 to 2 per cent. admixture of ceria. This is placed over a non-luminous Bunsen flame, the heat of which renders the mantle powerfully incandescent. The function of the gas is to generate heat, and that of the mantle is to transform the non-luminous heat energy into luminous light radiation.

It is curious that neither a mantle of pure thoria nor one of pure ceria is at all efficient for light emission, and the wonderful incandescence which is the result of adding less than 2 per cent. of ceria to a thoria mantle is difficult of explanation. It is frequently ascribed to a catalytic action causing great local intensity of combustion somewhat similar to that which induces the union of oxygen and hydrogen in the pores of spongey platinum.

Doubt seems to be thrown on this theory, however, by the fact, established by Le Chatelier, that the light emitted by the mantle is the same whether it is heated in the hot gases at the required temperature before or after they are burnt. Another explanation which commends itself to the writer is based upon the fact that the emitted waves of a pure thoria mantle are largely ultra-blue, or too fast for vision, and those from a ceria mantle ultra red, or too slow. It is suggested that the admixture of ceria so loads the thoria particles as to tune down their vibrations into the bright range of the visible spectrum, and thus to make evident to the eye rays which would otherwise be invisible.

To whatever cause it may be due, the emissive power of the Welsbach mantle is certainly remarkable. The ordinary C mantle, which has been much improved during the last five years, and which is now in general use, frequently gives as much as 70 candle power for a consumption of 3·8 cubic feet of London gas, or an "efficiency" of over 18 candle power per cubic foot of gas burnt per hour. The writer has lately obtained from an ordinary C mantle the high efficiency of 20 candle-power per cubic foot. And this is not by any means the best that can be done, for a new type of Welsbach burner is now coming on the market which, by a special construction, causes the gas to induce and thoroughly mix itself with almost the exact amount of air required for perfect combustion, and this without the aid of a chimney; thus a perfect Bunsen flame is produced, and very high incandescence obtained.

The writer's laboratory is at present lighted by one of these new burners, which is giving a light of 110 standard candles at an expenditure of 4·8 cubic feet of gas per hour, an efficiency ten times as great as that of good ordinary "fish-tail" or "batswing" burners. This means that, with South Metropolitan gas at 2s. 3d. per 1,000 ft., he is getting light at the rate of 110 candles for nearly seven and three-quarter hours for a pennyworth of gas.

In a practical estimate of the cost of Welsbach lighting under everyday conditions it will be necessary to make some deduction from the figures above quoted, and to allow, not only for renewal of mantles, but for a gradual decline in their illuminating power. Assuming a lamp to be lighted for 1,500 hours per year, it is a good rule to allow for three mantles for the period, and to take the average life at 500 hours. A mantle will generally be efficient after much longer use (the writer has one which is still efficient and has been in daily use for five years, since September 27th, 1893), but the too long use of a mantle is not always an economy. During the 500 hours' life it is safe, in the writer's experience, to expect an average efficiency of quite 14 candles per cubic foot of gas, so that we may rely upon getting a mean of 50 candle power at the cost of 3·6 cubic feet of gas per hour.

At the Yarrow Convalescent Home, Broadstairs, of which the writer is a Trustee, the building was originally planned for an electric installation, but inquiry having shown that the use of the incandescent gas light would result in a large annual saving, the electric scheme was abandoned, the engine, dynamo, and accumulator rooms were otherwise utilised, and 156 Welsbach burners were fitted.

During three years to September 30th, 1898, accident and decay of the mantles have been covered by 704 renewals, or less than two per burner per annum. During the same period the expenditure on other upkeep was less than 4d. per burner per annum.

The dining hall for 100 children and attendants, which is 64 by 27 square feet in floor area, has four triple gas pendants with 12 Welsbach lamps and "Cosmos" opal shades. Three burners are found to illuminate the whole hall sufficiently for ordinary purposes, and usually no more are lighted. The economy and general results of the Welsbach lighting have given great satisfaction to the Trustees.

Careful domestic arrangements have probably made these results exceptionally favourable, and, therefore, in the general estimate of the comparative cost of gas and electric domestic lighting which follows, a somewhat larger cost of upkeep is assigned to the Welsbach system than has been incurred at the Yarrow Home.

London experience of electric lighting, where the current is retailed at about 6d. per unit (1,000 watt-hours) shows that a glow lamp absorbing 64 watts gives, when new, a maximum light of 16 candles. In consequence, however, of the gradual deposit of carbon from the filament upon the glass, the light value falls off and an average of 14 candles is as much as should be expected during the useful life of a nominal 16 candle glow lamp. We will give the advantage, however, to the electric lamp and credit it with 16 candles.

When treating of the old system of open gas lighting it is assumed that the best No. 5 "Bray" burners are used, giving, with London gas, an efficiency of 2½ candle power per cubic foot. This is a very generous estimate, the average efficiency of open burners in practice being more commonly 1½ to 2, and the smaller sizes even less. With this explanation the following table will give the relative cost at London prices of lighting an institution requiring a total light of 4,000 candle power for 1,500 hours annually with electric incandescents, Bray burners, and Welsbach lamps.

Comparative Estimate.—Annual Cost of Maintaining Lights of 4,000 Total Candle power for 1,500 hours by Electricity and Gas.

| | | | |
|---|---|---|---|
| **Electric Incandescent—** | | | |
| 250 16-candle lamps, each absorbing 64 watts; cost of current at 6d. per unit ... ... | £600 | 0 | 0 |
| Renewal of lamps, 500 per annum, at 1s. 6d. each | 37 | 0 | 0 |
| Total ... ... ... ... ... | £637 | 0 | 0 |
| **Gas, Open Burners—** | | | |
| 320 Bray's "Special" burners, each giving 12½ c.p. for 5 feet of gas per hour; cost of gas, at 2s. 6d. per 1,000 cubic feet ... ... | 300 | 0 | 0 |
| Total ... ... ... ... ... | £300 | 0 | 0 |
| **Welsbach Incandescent—** | | | |
| 80 "C" burners, each giving 50 c.p. for 3·6 cubic feet of gas per hour; cost of gas ... ... | 54 | 0 | 0 |
| Renewal of mantles (240) and extra upkeep, 3s. 6d. per burner per annum... ... ... | 14 | 0 | 0 |
| Total ... ... ... ... ... ... | £68 | 0 | 0 |

Economies can, of course, be effected in electric lighting by the use of small lamps (8 candle) in places where only a little light is required, and also by making the most of the facility afforded for turning the current on and off as required. These advantages are almost entirely met in the Welsbach system by the new small lamps now being supplied, which burn only 1 to 1½ cubic feet per hour, and by the almost universal use of the by-pass burner.

Making all due allowance, however, for some advantage which electric lighting certainly possesses in these and other respects, we cannot help being impressed by the broad deduction from the above table, that electric lighting is twice as dear as the old gas system, and nine times as dear as the Welsbach.

When the new incandescent burners are commonly adopted, the advantage of the Welsbach will be further enhanced.

(*To be continued.*)

